# Emerging Therapies and Future Directions in Targeting the Tumor Stroma and Immune System in the Treatment of Pancreatic Adenocarcinoma

**DOI:** 10.3390/cancers10060193

**Published:** 2018-06-11

**Authors:** Daniel H. Ahn, Ramesh K. Ramanathan, Tanios Bekaii-Saab

**Affiliations:** Department of Hematology/Medical Oncology, Mayo Clinic Cancer Center, 5777 E. Mayo Blvd, Phoenix, AZ 85054, USA; ahn.daniel@mayo.edu (D.H.A.); ramanathan.ramesh@mayo.edu (R.K.R.)

**Keywords:** desmoplasia, microenvironment, pancreatic adenocarcinoma, stroma, novel targeted therapies, immunotherapy

## Abstract

Pancreatic adenocarcinoma is typically refractory to conventional treatments and associated with poor prognosis. While therapeutic advances over the past several years have improved patient outcomes, the observed benefits have been modest at best, highlighting the need for continued development of alternate treatment strategies. The tumor microenvironment has been identified as being integral to oncogenesis through its direct effect on cellular pathway communication, immune inhibition, and promoting chemo-resistance. A more in depth understanding of the biology of the disease, in addition with our ability to develop more effective novel therapies have led to ongoing studies that are investigating several promising treatment options in this disease. Herein, we highlight and review the therapeutic landscape in pancreatic adenocarcinoma.

## 1. Introduction

**Figure 1:**
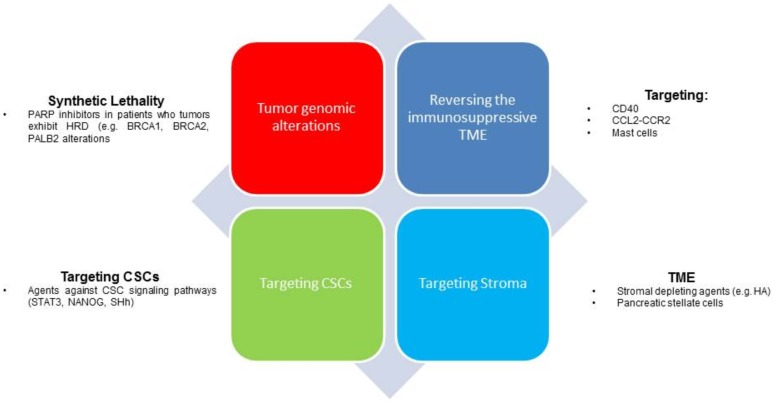
Treatment strategies in pancreatic adenocarcinoma. The figure provides an overview of novel treatment strategies in treatment of PDA. HRD, homologous recombinant deficiency; HA, hyaluronic acid; CSCs, cancer stem cells; SHh, Sonic Hedgehog pathway; MET, tumor microenvironment.

In the United States, pancreatic ductal adenocarcinoma (PDA) is the fourth leading cause of cancer related deaths, with a five-year survival rate of less than 10%, it will become the second leading cause of cancer-related deaths in this country by 2030 [1]. In 2017, roughly 53,670 new cases of pancreas cancer were diagnosed, with approximately only 10% of patients with clearly resectable disease at presentation [1,2]. During the past decade, therapeutic advances include the approval of two chemotherapy regimens for patients with newly diagnosed metastatic PDA and the first approved chemotherapy regimen for those with treatment refractory disease [3,4,5]. Despite these advances, patient outcomes remain dismal where the median overall survival remains less than 12 months, underscoring the continued investigation and need for the development of novel therapies in this disease. An increased understanding of the pancreatic tumor microenvironment includes the tumor stroma and its involvement in chemotherapy resistance and immunosuppression. This has resulted in the development of novel therapies to overcome these tumor intrinsic factors. Herein, we review and highlight novel therapeutic approaches aimed at the tumor microenvironment and strategies to overcome the immunosuppression in PDA (Figure 1).

## 2. Targeting Pancreatic Cancer Stroma

### 2.1. Tumor Stroma

The histologic feature observed in PDA that is integral to its innate characteristic features is the tumor microenvironment (TME). TME is comprised of cellular elements and a marked desmoplastic reaction. TME is comprised of various cell types that include mesenchymal cells, which are most notably fibroblasts of various types including pancreatic stellate cells (PSCs), inflammatory associated cells, glycoasminoglycans, and the vascular endothelium [6]. PSCs are a subset of pancreatic cancer associated fibroblasts that are integral in tumor-stromal interactions and the development and maintenance of the desmoplasia. PSCs are resident cells of the pancreas that remain in a quiescent state and function to regulate normal tissue architecture [7]. In PDA, pancreatic cancer cells activate PSCs through various signaling (MAPK, PI3k/Akt, and JAK-STAT) pathways, inflammatory cytokines and reactive oxygen species that induce aberrant activation of PSCs [8]. Activated PSCs can produce autocrine factors (TGFβ1, PDGFR, IL-1, IL-6, etc.), which further potentiate its activity that results in the synthesis of excessive extracellular matrix (ECM) ECM proteins, secretion of growth factor and cytokines that induce pancreatic cancer cell growth and migration [9]. The overproduction of ECM contributes to the fibrotic reaction that contributes to tumor proliferation and chemotherapy resistance in PDA [10]. PSCs also induce endostatin production from pancreatic cancer cells, which contributes to intratumoral hypoxia, which serves as another mechanism for chemotherapy resistance [11]. Thus, the fibrotic and avascular microenvironment decreases effective chemotherapy delivery, which contributes to treatment resistance and tumor progression.

PSCs also help mediate the immunosuppressive TME associated with PDA. In preclinical human PDA samples demonstrated that activated PSCs reduced the CD8^+^ T cell migration within the tumor stroma [12]. Furthermore, further work has shown PSCs promote the differentiation of immune inhibiting myeloid-derived suppressor cells, suggesting PSC’s contribute to the immunosuppressive TME observed in PDA [13].

Given the various roles that PSCs serve in contributing to tumor proliferation and treatment resistance in PDA, there is significant interest in various strategies aimed at targeting PSCs, which including inhibiting PSC proliferation and their conversion into an inactive quiescent state. While these strategies are intriguing, fibroblasts including PSCs play a protective role (below) and its inhibition can potentially result in PDA proliferation.

### 2.2. Hyaluronic Acid

Hyaluronic acid (HA), is one of several glycoasminoglycans that comprises the ECM and is present in normal tissue in several organs. HA is a glycoasminoglycan commonly found in the extracellular matrix and high HA content in observed across several solid tumors including pancreas cancer. In pancreas cancer, high HA content is present as high as 90% of PDA tissue and is a poor prognostic factor [14]. Chemoresistance is thought to be in part due to the ECM, where HA polymers bind and trap water molecules to alter the tissue architecture into a mechanical barricade that impedes effective chemotherapy delivery to neoplastic cells. HA also binds to several cell surface receptors to activate downstream cell signaling pathways associated with tumor proliferation and treatment resistance [14]. While ECM is thought to act as a barrier or impedance to effective chemotherapy, additional works suggest that it may also serve an integral structure role where its manipulation can revert PDA to a more aggressive biologic disease [15,16].

Given the association between HA and tumor proliferation and chemotherapy resistance, preclinical work have explored the exploitation of HA as a potential target in the treatment of pancreas cancer. PEGylated hyaluronidase α (PEGPH20; Haylozyme Therapeutics) is a pegylated form of recombinant hyaluronidase, which lengthens the circulatory half-life (>20 h) to augment HA stromal degradation [17]. In preclinical animal studies, PEGPH20 normalized intra-tumor interstitial fluid pressures, enhanced intra-tumor chemotherapy delivery and tumoricidal activity [18]. Consistent with preclinical findings, observations from the HALO-109-101 and HALO-109-102 phase 1 trials, PEGPH20 increased plasma HA and tumor permeability, while decreasing tumor metabolic activity [19]. These results led to the randomized phase 2 trial, where patients with treatment naïve metastatic PDA received gemcitabine/nab-paclitaxel alone or in combination with PEGPH20 [20,21] (Table 1). Correlative work included baseline tumor HA level assessment, where HA levels were categorized as HA^low^ or HA^high^, which was defined by HA staining in the ECM as <50% or ≥50% of tumor surface, respectively. In the first stage, 135 patients were randomized to receive gemcitabine/nab-paclitaxel alone or in combination with PEGPH20. Similar progression free survival (PFS) were observed between the two treatment arms (5.7 vs. 5.2 months, HR = 0.69, *p* = 0.11) with an increase of thromboembolic events in the PEGPH20 arm (43% vs. 25%) [20]. In the 47 patients with HA^high^ tumors, a significant improvement in PFS was observed in patients who received PEGPH20 (9.2 with PEGPH20 vs. 5.2 months, (HR, 0.45; 95% CI, 0.17 to 1.22; *p* = 0.11) [21]. Based on these results, in stage 2 of the trial, an additional 133 patients were randomized in a 2:1 fashion to receive gemcitabine/nab-paclitaxel in combination with PEGPH20 (PAG) or gemcitabine/nab-paclitaxel (AG) alone [22]. The two primary end points of the study were overall PFS (stages 1 and 2) and incidence of thromboembolic events in stage 2. The study’s co-primary endpoints were met. 84 (34%) of the 246 patients (with evaluable HA data) were identified as having HA^high^ tumors. A nominal but statistically significant improvement in PFS was seen in the PAG arm (6 vs. 5.3 months; HR 0.73; *p* = 0.049) in the protocol-defined efficacy evaluation population. The benefit was more pronounced in patients with HA^high^ tumors. Empiric prophylactic enoxaparin in the stage 2 of the study resulted in similar thromboembolic events between the two treatment arms (grade ≥ 3 bleeding events, 4% in the AG arm vs. 8% PAG). These results led to HALO 301, an ongoing international randomized phase 3 trial, where patients with treatment naïve metastatic pancreatic adenocarcinoma with HA^high^ tumors are randomized in a 2:1 fashion to receive gemcitabine/nab-paclitaxel alone or in combination with PEGPH20 [23]. In contrast, S1313, a randomized phase 1b/2 trial that investigated FOLFIRINOX cytotoxic chemotherapy in combination with PEGPH20, was halted for futility based off the planned interim analysis [24]. The median overall survival in the FOLFIRINOX arm was 14.4 months vs. 7.7 months in the PEGPH20 arm, favoring the standard arm (HR 0.48; *p* < 0.01) [24]. These findings suggest a potential detrimental effect with the addition of PEGPH20 to FOLFIRINOX in patients with metastatic pancreas cancer. Preclinical studies provide insight to the observed S1313 results, as stroma depletion induced a more biologically aggressive form of pancreas cancer through VEGF dependence and enhanced immunosuppressive effects [15,16]. The unexpected S1313 results support that further work, including an evaluation to explain the observed findings, is needed prior to its development in future studies.

### 2.3. Sonic Hedgehog Pathway (SHh)

The SHh is a signaling pathway that transmits information to embryonic stem cells for cell differentiation and organogenesis. It is often inactive in adult tissues but also regulates adult stem cells and is involved in tissue preservation [25]. SHh overexpression is observed in various malignancies including PDA, where it is integral to the development of the paracrine signaling network that promotes desmoplasia formation [25,26]. Cancer associated fibroblasts, an integral component of the pancreas stroma, have also been noted to exhibit aberrant SHh activity [27].

In pancreas cancer mouse models, IPI-926, a SHh inhibitor, resulted in increased gemcitabine delivery by depleting stromal tissue and increasing vascular density [28]. Unfortunately, the promising preclinical activity did not translate to an improvement in patient outcomes in clinical trials. A phase 2 trial investigated gemcitabine monotherapy or in combination with IPI-926 in treatment naïve patients with metastatic PDA. Patients who received the combination of gemcitabine and IPI-926 experienced an inferior PFS and OS when compared to the control arm [29]. Another phase 2 randomized trial in patients with treatment naïve metastatic PDA also failed to demonstrate an improvement in PFS in patients that received the combination of gemcitabine with vismodegib, another SHh inhibitor [30]. The discordance seen between the preclinical and observed outcomes in clinical trials is unclear. More recent preclinical work suggests a potential detrimental effect from SHh inhibition, where increased IPI-296 exposure led to a more aggressive phenotype with undifferentiated histology, increased tumor cell proliferation and vascularity [15]. Similar results were also observed in preclinical studies with vismodegib [31]. Thus, it is feasible that certain components within the tumor microenvironment serve a protective purpose by restraining tumor growth and metastases. While the results from a completed phase 1 study with IPI-926 in combination with FOLFIRINOX in PDA have suggested potential clinical activity [32], the cumulative results observed with this group of agents suggest that the continued investigation of SHh inhibitors in pancreas cancer is unlikely to be yielding any meaningful efficacy.

## 3. The Promise and Challenge of Immune Targeting in PDA

While immuno-oncologic agents have shown meaning clinical activity across several solid tumor malignancies, results from early clinical trials in PDA have been disappointing [34,35]. Its unique tumor microenvironment (TME) promotes immune evasion by suppressing tumor infiltrating lymphocyte activity (TILs) that contribute to dampening an anti-tumor immunogenic response [36]. Thus, strategies aimed at overcoming immunosuppression activity, which include agents that inhibit T-cell immune checkpoints with anti-PD1 or anti-CTLA-4 monoclonal antibodies, have not translated to clinical activity in pancreas cancer [34,37]. The identification of alternative targets within the TME may result in the identification and development of more effective immunotherapeutic agents in this disease.

### 3.1. CD40

CD40 is a co-stimulatory protein present on antigen presenting cells (APCs), which CD4^+^ T helper cell activation requires the presence of CD40. Activated APCs are required to convert CD8^+^ T cells into cytotoxic effector T cells. Thus, through an indirect effect, CD40 activating monoclonal antibodies activate CD8^+^ T cells and potentially can reverse the immunosuppressive environment observed in pancreas cancer [38]. In early studies, the concomitant administration of CD40 agonist antibodies with gemcitabine resulted in clinical responses in PDA patients, where 4 of 21 patients experienced a partial response per RECIST criteria [39]. Moreover, correlative studies confirmed that CD40 activated macrophages induced the observed anti-tumor activity [39]. These results suggested that the observed immunogenic tumoricidal activity was derived from a CD40 dependent mechanism and potentially represent another treatment approach against PDA. In a phase 1 study, CP-8970,893, a CD40 agonist antibody, was given in combination with gemcitabine in patients with treatment naïve unresectable PDA [40]. Four of the 22 enrolled patients experienced a partial response, which demonstrated the potential of CD40 agonists as a treatment modality in pancreas cancer. An ongoing phase 1/2 study is investigating APX005M, a CD40 agonistic monoclonal antibody with gemcitabine/nab-paclitaxel chemotherapy and the addition of Nivolumab, an anti-PD-1 monoclonal antibody [41].

### 3.2. CCL2-CCR2

The CCL2-CCR2 signaling axis has culminated enthusiasm as a novel therapeutic target in PDA. Chemokine CCL2 binding to CCR2, its cognate receptor, elicits the recruitment of monocytes and tumor-associated macrophages (TAMs) into the tumor microenvironment. These cells contribute to immunosuppression while inducing metastases, immune evasion and chemotherapy resistance. In preclinical studies, CCL2 production induced CCR2^+^ TAMs infiltration [42]. Coinciding clinical outcomes also revealed tumors that displayed high CCL2 expression/low CD* T-cell infiltrate was a poor prognostic factor. In PDA mouse models, CCR2 inhibition depleted inflammatory monocytes and macrophages, which resulted in enhanced chemotherapy efficacy and anti-tumor T-cell response while inhibiting tumor cell growth and metastasis [42,43]. A recently completed randomized phase 1B trial evaluated FOLFIRINOX in combination with PF-04136309, a CCR2 inhibitor, in patients with borderline resectable or locally advanced pancreatic adenocarcinoma. Patients who received the combination experienced a 48.5% response rate in comparison to the pre-specified expected response rate of 25% with FOLFIRINOX monotherapy [44]. Another randomized phase 1b/2 clinical trial investigated the combination of gemcitabine/nab-paclitaxel with PF-04136309 has also been completed with pending results [45]

### 3.3. Targeting Mast Cells

The characteristic desmoplastic stroma in PDA is composed of inflammatory cells, which include mast cells that function as an integral component within the tumor microenvironment. Comprehensive assessment of human PDA samples revealed that mast cell infiltration was associated with poor prognostic factors that included higher tumor grade and worse overall survival [46], while increased mast cell concentration correlated with lymphatic and microvascular invasion and lymph node metastasis [47]. Ibrutinib, a Bruton’s tyrosine kinase (BTK) inhibitor, inhibits mast cell degranulation and has been approved for the treatment of chronic lymphocytic leukemia and mantle cell lymphoma [48,49]. In PDA mouse models, Ibrutinib resulted in decreased tumor-associated inflammation and fibrosis, suggesting that PDA associated fibrosis is a mast cell dependent phenomena [50]. Ibrutinib also exhibited an anti-tumor effect through its inhibition of pancreatic cancer growth and increased gemcitabine cytotoxicity while enhancing T cell-dependent immune related tumoricidal activity [50,51]. These results lead to RESOLVE clinical trial, a randomized phase 2/3 trial that is investigating gemcitabine/nab-paclitaxel alone or in combination with Ibrutinib, which has completed accrual. The primary endpoint of the study is PFS and the results will become available soon [52]. 

## 4. Synthetic Lethality: Targeting Deficiencies in Homologous Recombination and DNA Repair

In PDA, approximately 15% of patients harbor germline genomic alterations that increase susceptibility to the development of solid tumor malignancies [53]. Of these genes, alterations were observed most often in genes in the DNA mismatch repair (MMR) pathway, including *BRCA1*, *BRCA2,* and *PALB2* [53]. In patients at risk, including individuals of Ashkenazi Jewish descent or those with a strong family history of pancreas cancer, the prevalence of *BRCA1* and *BRCA2* germline mutations has been reported in up to 19% [54,55].

*BRCA1/2* and *PALB2* play an integral role in homologous recombination and DNA damage response [56]. Alternatively, PARP (poly (ADP)-ribose polymerase), a family of proteins that function to detect and initiate single-strand DNA break repair in the setting of BRCA dysfunction. PARP is a critical enzyme of cellular proliferation and mediates DNA repair of DNA single strand breaks and rescues tumor cells from DNA damage [57]. Thus, agents that inhibits PARP represents a rationale treatment strategy for patients with PDA who tumors harbor alterations in the MMR pathway. In the setting of deficient homologous repair and the inability to induce efficient DNA repair, tumors may have an enhanced sensitivity to DNA damaging cytotoxic chemotherapeutic agents that include platinum analogues and PARP inhibitors [58,59]. In several retrospective case series, patients with *BRCA1/2*-associated PDA achieved an improvement in objective response and OS when receiving platinum-containing chemotherapy regimens [60,61]. In the setting of homologous recombination deficiency, PARP inhibition can restrict the repair of these single-strand DNA breaks, resulting in DNA double-strand breaks and cell death. Several PARP inhibitors have been investigated across clinical trials in patients with BRCA mutant tumors with differing results. A phase 2 trial examined olaparib, an oral PARP inhibitor, in patients with treatment refractory germline *BRCA1/2* mutant tumors [62]. The primary endpoint of the study was response rate. In the 23 patients with PDA, 22% objective response was observed, with an additional 35% of patients having stable disease, consistent with findings seen in other solid tumor malignancies [62]. RUCAPANC, an open-label phase 2 trial investigated rucaparib, an oral PARP inhibitor, in previously treated patients with *BRCA1/2* mutant pancreatic adenocarcinoma [63]. Patients experienced a 11% response rate (in addition to one unconfirmed response), where the duration of confirmed responses was ongoing at 36 and 49 weeks [63]. In contrast to the two previously mentioned studies, in a phase 2 trial, no confirmed responses were observed with veliparib an oral PARP inhibitor, in individuals with treatment refractory *BRCA1/2* or *PALB2* mutant pancreatic adenocarcinoma [33]. The differences in observed anti-tumor activity from PARP inhibition is likely due to the differing efficacy of each PARP inhibitor rather than class activity of PARP inhibitors in homologous recombinant deficient PDA. From the enhanced tumoricidal activity observed with PARP inhibitors in the setting of existing DNA damage, PARP inhibition in combination with DNA damaging cytotoxic chemotherapy represents another strategy of interest. An ongoing randomized phase 2 study in patients with treatment refractory PDA is assessing FOLFIRI systemic chemotherapy alone or in combination with veliparib, with OS as its primary endpoint [64]. In conjunction with ongoing treatment strategies with PARP inhibitors, another area of interest is in their potential as maintenance therapy in patients whose tumors exhibit homologous recombinant deficiency. POLO (Olaparib in gBRCA Mutated Pancreatic Cancer Whose Disease Has Not Progressed on First Line Platinum-Based Chemotherapy), an ongoing randomized phase 3 study, is investigating olaparib in patients with metastatic *BRCA1/2* mutant pancreas cancer as a maintenance strategy following platinum based chemotherapy [65].

## 5. Targeting Cancer Stem Cells

Cancer stem cells (CSCs) are typified by their ability to produce tumor cells with varying phenotypes. Through activation of anti-apoptotic pathways and increased DNA repair mechanisms, CSCs are resistant to chemotherapy and radiotherapy. Furthermore, the exposure to conventional cytotoxic therapies can elicit “stemness” in cancer cells where CSCs are able to convert non-CSCs to CSC-like cells. CSC-like cells are able to persist after treatment and may serve as a mechanism for relapse after therapy. While uncertainty exists about true nature of CSCs and their role cancer proliferation, in PDA, CSCs have been identified as being CD44^+^CD24^+^ESA^+^, CD133^+^/CXCR4^+^, or ALDH^high^CD44^+^CD24^−^ cells [66,67,68], where these cells are highly tumorigenic, promote metastatic spread, and are associated with poor survival. Since CSCs are resistant to traditional therapies including gemcitabine chemotherapy and radiotherapy [61,62], targeting the signaling pathways (Hedgehog, NANOG, STAT3) that drive cancer cell stemness represent a strategy against CSCs.

### 5.1. Wnt/β-Catenin

Wnt pathway activation occurs in up to 65% of patients with PDA, and is important for CSC renewal, cell differentiation, tumorigenicity and epithelial-mesenchymal transition, which is in part responsible for resistance to conventional DNA damaging therapies (chemotherapy, radiotherapy). Wnt pathway is a key CSC signaling pathway that regulates CSC survival and proliferation. In a phase I trial of OMP-54F28 (OncoMed Pharmaceuticals, Redwood City, CA, USA) in patients with solid tumors, an Wnt pathway antagonist, 3 out of 26 patients experienced stable disease for >6 months [69]. In combination with gemcitabine in patients with treatment refractory PDA, a median PFS of 2 months was observed. Currently, other Wnt inhibitors that are under early investigation include CGX1321 [70] and a recently completed phase IB trial with Vantictumab in combination with gemcitabine/nab-paclitaxel in patients with previously untreated metastatic PDA [71].

### 5.2. JAK/STAT

JAK family receptor activation results in phosphorylation of STAT transcription factors. In PDA, JAK2/STAT3 activation contributes to cell cycle progression, anti-apoptosis and angiogenesis [72]. In KRAS mutant PDA mouse models, STAT3 inhibition resulted in tumor volume reduction and decreased cancer cell proliferation [73]. While early phase trials with JAK/STAT inhibitors showed promising results, in phase 3 trials, this did not translate to an improvement in patients’ outcomes in PDA. JANUS-1, a randomized phase 3 trial, investigated the combination of Ruxolitinib, a janus kinase inhibitor selective for JAK1 and JAK2, with capecitabine in patients with treatment refractory metastatic PDA in patients with CRP (C-Reactive Protein) > 10. Unfortunately, at the interim analysis, the study was discontinued for futility [74].

Inhibition of STAT3 transcription is another approach that has demonstrated promising results in targeting “stemness” in PDA. Napabucasin (BBI-608, Boston Biomedical Inc., Boston, MA, USA) an orally available first-in-class cancer stem cell inhibitor that appears to target and inhibit gene transcription induced by STAT3 and cancer cell stem cell properties, demonstrated the ability to inhibit relapse in PaCa-2 PDA xenograft mouse models [75]. This preliminary activity resulted in the investigation of napabucasin in a phase 1b/2 trial in metastatic PDA. Patients received Napabucasin 240 mg twice daily with weekly gemcitabine/nab-paclitaxel until disease progression or other criteria for discontinuation [76]. Among the 60 patients with measurable disease who were enrolled in the study, disease control, which was defined as complete response + partial response + stable disease, was observed in 55 patients (92%) with two complete responses (3.3%) and 26 partial responses (43%) [76]. Treatment was well tolerated, where most treatment related adverse events were grade 1 and 2 gastrointestinal events. Grade 3 adverse events were observed in 12 patients, primarily due to fatigue (8) and gastrointestinal symptoms (3) [76]. Based on these results, CANSTEM 111p [77], an ongoing randomized phase III trial is investigating gemcitabine/nab-paclitaxel monotherapy or with napabucasin to confirm the clinical activity observed in the phase 1b/2 study.

## 6. Conclusions and Future Directions

At this present time, the treatment of pancreatic adenocarcinoma remains a challenge, with limited treatment options that provide modest improvements in patient outcomes. Despite advances that include the recent molecular characterization of PDA, and an increased understanding of the TME and its role in chemotherapy resistance, direct usage of the knowledge has not translated into an improvement in patient outcomes, where treatment options remain limited to cytotoxic chemotherapy regimens (FOLFIRINOX, gemcitabine/nab-paclitaxel, Onyvide). Nevertheless, further advances are needed, and several promising treatment strategies are being investigated and outlined above. These efforts include utilizing tumor genomic profiling to identify the subset of patients with deficiencies in homologous recombination and DNA repair that will likely benefit from novel agents aimed at exploiting these defects (e.g., PARP inhibitors, platinum chemotherapy agents). Furthermore, while several studies have not demonstrated success in targeting the tumor stroma, further refinement and an ongoing phase 3 trial will inform us if this remains a relevant treatment strategy in PDA. Additional alternative therapeutic approaches include novel agents aimed at targeting cancer stem cell properties, immune sensitization, and the tumor microenvironment. These innovative approaches have reignited optimism and enthusiasm to spur investigation in both early- and later-phase clinical trials that hopefully will result in improving patient outcomes in this terrible disease.

## Figures and Tables

**Table 1:** Summary of ongoing or completed clinical trials investigating novel therapeutic agents in pancreatic ductal adenocarcinoma.

Agent	Phase	Primary Endpoint	Treatment	Median PFS	Median OS	Comments	References/NCT *
PEGPH20	2	PFS	AG vs. PAG	5.7 vs. 5.2 mos, HR = 0.69, *p* = 0.11	Pending	TE events (25% vs. 42%)	[20]
PEGPH20	2	PFS	AG vs. PAG	9.2 vs. 5.2 mos	Pending	TE events similar (PAG 14% vs. AG 10%)	[22]
PEGPH20	2	PFS	FOLFIRINOX ± PEGPH20	Pending	Pending	Halted early due to futility	NCT01959139
APX005M	1/2	Safety, tolerance, PFS	PX005M + Gemcitabine/Nab-paclitaxel ± Nivolumab	Pending	Pending		NCT03214250
PF-04136309	1b/2	Safety, tolerance, PFS	PF-04136309 + Gemcitabine/Nab-paclitaxel	Pending	Pending	Treatment naïve	NCT02732938
Ibrutinib	2/3	PFS	Ibrutinib + Gemcitabine/Nab-paclitaxel	Pending	Pending		NCT02436668
Napabucasin	3	OS	Gemcitabine/Nab-paclitaxel ± Napabucasin	Pending	Pending		NCT02993731
Veliparib	2	OS	FOLFIRI ± Veliparib	Pending	Pending		NCT02890355
Olaparib	3	PFS	Olaparib vs. Placebo	Pending	Pending	Germline *BRCA1/2* mutations; in pts whose have not progressed on 1st line platinum chemo	NCT02184195
Rucaparib	2	ORR	Rucaparib (single arm) in *BRCA1/2* mutant patients	Not available	Not available	11% ORR including 1 CR. Duration of confirmed responses at 36 and 49 weeks	NCT02042378
Veliparib	2	ORR	Veliparib (single arm) in *BRCA1/2* mutant patients	1.7 mos	3.1 mos	No responses	[33]

AG—gemcitabine/nab-paclitaxel; PAG—PEGPH20 + gemcitabine/nab-paclitaxel; mos—months; TE—thromboembolic; PFS—progression free survival; OS—overall survival; ORR—objective response rate. * http://clinicaltrials.gov.
